# Advance directives in the emergency department–a systematic review of the status quo

**DOI:** 10.1186/s12913-024-10819-1

**Published:** 2024-04-03

**Authors:** Vincent Weber, Aurelia Hübner, Sandra Pflock, Lukas Schamberger, Rajan Somasundaram, Lennert Boehm, Wolfgang Bauer, Eva Diehl-Wiesenecker

**Affiliations:** 1 Department of Emergency Medicine, Charité - Universitätsmedizin Berlin, Corporate Member of Freie Universität Berlin and Humboldt-Universität zu Berlin, Berlin, Germany; 2 Wollongong Hospital, Illawarra Shoalhaven Local Health District, Wollongong, NSW Australia; 3 Emergency Department, Medical Faculty, University Hospital Düsseldorf, Heinrich Heine University, Düsseldorf, Germany

**Keywords:** Advance directives, Palliative care, Emergency department, hospital, End of life decisions, Resuscitation orders

## Abstract

**Background:**

Providing individualised healthcare in line with patient wishes is a particular challenge for emergency healthcare professionals. Documentation of patient wishes (DPW), e.g. as advance directives, can guide clinicians in making end-of-life decisions that respect the patient’s wishes and autonomy. However, patient centered decisions are hindered by limited availability of DPWs in emergency settings.

**Objective:**

This systematic review aims to congregate present data on recorded rates for DPW existence and availability in the emergency department (ED) as well as contributing factors for these rates.

**Methods:**

We searched MEDLINE, Google Scholar, Embase and Web of Science databases in September 2023. Publications providing primary quantitative data on DPW in the ED were assessed. Publications referring only to a subset of ED patients (other than geriatric) and investigating DPW issued after admission were excluded.

**Results:**

A total of 22 studies from 1996 to 2021 were included in the analysis. Most were from the US (*n* = 12), followed by Australia (*n* = 4), Canada (*n* = 2), South Korea, Germany, the United Kingdom and Switzerland (*n* = 1 each). In the general adult population presenting to the ED, 19.9–27.8% of patients reported having some form of DPW, but only in 6.8% or less it was available on presentation. In the geriatric population, DPW rates (2.6–79%) as well as their availability (1.1–48.8%) varied widely. The following variables were identified as positive predictors of having DPW, among others: higher age, poorer overall health, as well as sociodemographic factors, such as female gender, having children, being in a relationship, higher level of education or a recent previous presentation to hospital.

**Conclusions:**

Existence and availability of a recorded DPW among ED patients was low in general and even in geriatric populations mostly well below 50%. While we were able to gather data on prevalence and predictors, this was limited by heterogeneous data. We believe further research is needed to explore the quality of DPW and measures to increase both rates of existence and availability of DPW in the ED.

**Supplementary Information:**

The online version contains supplementary material available at 10.1186/s12913-024-10819-1.

## Background

Every medical intervention requires the patient’s consent– both potentially curative as well as palliative measures. However, in case of an emergency treatment the patient’s capacity to understand and consent to or decline an intervention can be impaired. In these situations, it can be advantageous for patients, their relatives and healthcare providers to have access to some form of prearranged documentation on the patient’s beliefs and wishes to guide treatment, summarized under the term Documentation of Patient´s Wishes (DPW).

Patients with life-limiting illness are often seen in the emergency department (ED) [1]: more than three-quarters of those aged ≥ 65 years will have a visit to the ED in the last six months of life [2]. Due to demographic change, the amount of patients requiring end-of-life care will likely continue to increase, including in emergency settings [3].

EDs often set the course for further treatment both in hospital as well as outpatient settings. Due to the need for fast decision-making in the care of patients with acute illness, evaluating goals of care can be difficult challenging and at times impossible, particularly when a patients’ decision-making abilities are impaired, and relatives or proxies are unavailable to help ascertain the patient’s likely wishes and preferences for goals of care [4]. Therefore, patients requiring palliative care or having special preferences, such as the refusal of blood transfusion for religious reasons, e.g., Jehovah’s Witnesses, are at risk of not having their therapeutic goals met in the emergency department [5, 6].

Thus, it is essential to sufficiently train health care providers in palliative medicine and end-of-life care [7, 8], identify patients’ needs in a standardized manner [1, 4, 9], and communicate their wishes efficiently and as concretely as possible– for example by means of DPW [10]. Available DPW upon presentation to the ED could preserve patient autonomy once decision-making capacity is lost, reduce the occurrence of procedures not in line with patient wishes and distribute ED resources more efficiently.

The scope of this review includes (1) Advance Directives (ADs) or Living Wills (LWs), wherein the patient declares which specific medical measures may be undertaken, (2) Advance Care Planning (ACP) documents as an extension of the original ADs, which provide a repeated evaluation of patients’ preferences concerning goals of care based on their wishes and values, and (3) the commonly used Do Not Resuscitate (DNR) and Do Not Intubate (DNI) orders, which can be put in place either verbally by patients themselves or via any of the aforementioned documents. Health Care Proxy or Power of Attorney documents, which delegate decisions in certain areas of life to other persons, are not included [5]. Furthermore, differences between AD and ACP [10], consideration of DPW by health care providers and its impact [11, 12] or initiation of palliative measures in the ED [12–15] are not explored in this paper.

This analysis aims to assess the status quo, focusing on general ownership of DPW in patients presenting to the ED, their availability to the treatment team on site and predictive factors influencing the likelihood of patients having a DPW.

## Methods

This systematic review was conducted using several databases and in accordance with the Preferred Reporting Items for Systematic review and Meta-Analysis and the according protocols (PRISMA and PRISMA-P) [16, 17]. The PRISMA-P is a reporting guideline which aims to provide a comprehensive protocol for systematic reviews to promote transparency of review methods [17].

### Data source and search

An electronic search was conducted through the online databases Cochrane, Web of Science, Embase via Ovid, Google Scholar and MEDLINE (PubMed) in September 2023 in accordance with recommendations for systematic literature searches by Bramer et al. to guarantee adequate and efficient coverage [18]. The search algorithms used are displayed in Supplementary, Tab. s1. The bibliographies of all included studies were screened for references to other original sources.

### Study selection and data extraction

We included studies that were (1) published before September 2023, (2) available in English or German, (3) conducted in an ED setting, (4) gathered primary quantitative data on existence and/or availability of DPW, and (5) investigated either general adult populations or geriatric populations defined by age, morbidity or nursing home (NH) residence. Publications were excluded if (1) they referred to only a subset of ED patients defined by specialty (e.g., only dept. of surgery), illness (e.g., only sepsis) or patient disposition (e.g., only out- or inpatient), or (2) DPW was issued after ED contact. Data extraction was performed manually according to predefined categories relevant to the above-mentioned status quo analysis as well as sample characteristics.

### Selection process and data management

We screened the titles and abstracts for inclusion and exclusion criteria as described above. Full-text manuscripts meeting the inclusion criteria were applicable. The data extracted were added to Microsoft Excel (Version 2016, Microsoft Cooperation) data sheets. Data were then exported to CSV file format to be analyzed and visualized using R in RStudio (Version 2023.09.0 + 463, Posit Software, PBC).

### Data items, data synthesis and outcomes

For all studies included finally, characterizing data on setting, methodology and study population (sample size, age, gender, inclusion/exclusion criteria) were gathered. A descriptive analysis comprising all included studies was performed. The outcome parameters, which were extracted individually for each study, were: existence of a recorded DPW, availability of DPW and possible predictors for both.

### Risk of bias assessment

For assessing possible risk of bias of each study, the methodological quality was assessed by two reviewers using the most appropriate tool: *Joanna Briggs Institute (JBI) Clinical Appraisal Checklist for Prevalence Studies* [19]. The tool uses four answers: “yes”, “no”, “unclear” and “not applicable” based on an eight-item-scale.

## Results

### Search results, overview and characteristics of included studies

**Fig. 1:**
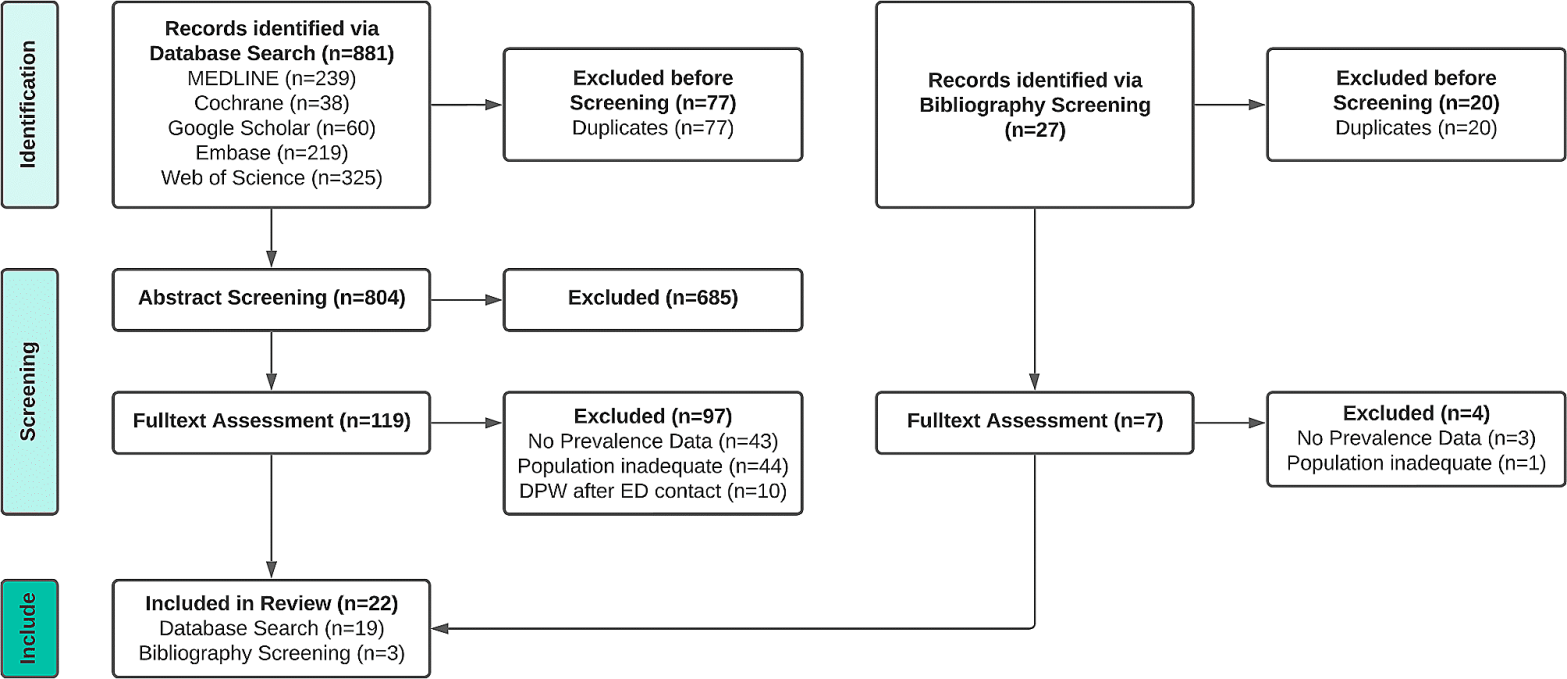
Flowchart representing search results, exclusion of studies due to applying the inclusion/exclusion criteria and final number of included studies

Our systematic database search identified 881 studies. Of the abstracts screened, *n* = 119 publications were retrieved for full-text assessment (excluded *n* = 685). 27 more were identified by screening the bibliographies of all included studies. After removing duplicates (*n* = 97) and applying the in- and exclusion criteria, a total of 22 studies were included in the analysis [4, 11, 20–38], whereas 104 studies (Supplementary, Tab. s2) were excluded (Fig. 1).

Of the 22 included studies, most (*n* = 12) were from the United States [4, 20–23, 27, 30–33, 37, 39], followed by Australia (*n* = 4) [11, 24, 29, 35], Canada (*n* = 2) [26, 38], South Korea [34], Germany [28], the United Kingdom [25] and Switzerland [36] (*n* = 1 each). The earliest study was published in 1996, the most recent in 2019, with over half (*n* = 13) of the research published after 2010. Almost all research was conducted in urban academic emergency departments, except for Davis et al. [27]. Lahn, Street and Kim were multi centric data studies [4, 29, 34]. Methodologically, all included studies were observational cross-sectional studies, using either surveys– oral or written– or analysis of patient records. 10 were performed prospectively, all others retrospectively (Supplementary Tab. s3).

In total, 33.664 ED patients participated across all studies. Median sample size was *n* = 300. Sample sizes were mostly in the range of *n* = 50 [39] to *n* = 1.131 [34], with the notable exception of Vranas et al. (*n* = 26.128) [37]. Women and men were represented almost equally with 51.31% of all subjects being female (not reported by Harrison [33]).

The included studies can be dichotomized by their inclusion criteria (geriatric population with age restriction of ≥ 65 years or presence of characteristic morbidities and/or nursing home residents vs. general adult population ≥ 18 years) as well as their research objective (DPW existence, availability or both of DPW in the ED) (Table 1).

Mean age among the studies classified as *adult population* varied from 49 to 66 years; in those classified as *geriatric population* from 76 to 86 years (given as median by several publications and thus not directly comparable; see Supplementary, Tab. s3).

The risk of bias assessment according to the JBI clinical appraisal checklist is depicted in Supplementary, Tab. s4. Questions 5–7 and mostly 9 were not applicable.

**Table 1:** Included publications by investigated research question and study population. Note, some studies investigated both existence and availability

	DPW Existence (*n* = 15)	DPW Availability (*n* = 17)
Adult Population	Llovera, Llovera, Slankamenac, Christ, Davis, Vranas	Slankamenac, Christ, Davis, Vranas
Geriatric Population	Taylor, Carter, Saliba, Gill, Platts-Mills, Street, Grudzen, Ishihara	Chua, Osman, Lahn, Kim, Wall, Gill, Platts-Mills, Street, Grudzen, Ishihara, Harrison, McQuown, Russell

### Existence of DPW

**Fig. 2:**
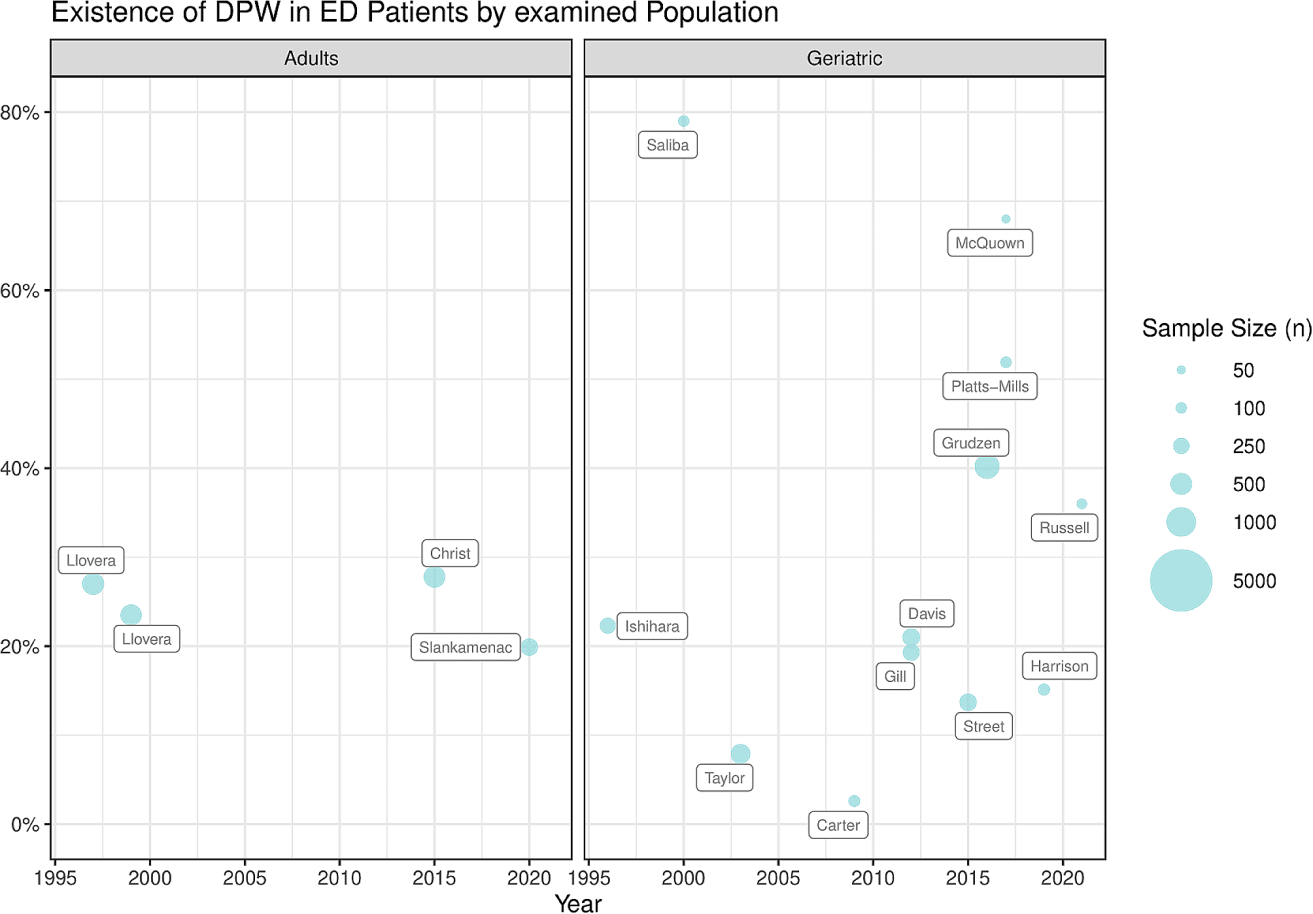
DPW existence by study population (left: adult, right: geriatric) and year of publication

The proportion of all ED patients having DPW was reported to range from 19.9% [36] to 27.8% [28], as described by five of the included studies [21, 22, 27, 28, 36] (Fig. 2). Slankamenac et al. conducted a follow-up after 60 days via e-mail, in which the rate of DPW had increased by 2.7% (loss-to-follow-up: 56.8%), simply due to having come into contact with the topic [36].

In geriatric populations, described by 11 studies, the variation in rate of DPW was greater, ranging from 2.6% [25] to 79% [23]. Only 4 studies were able to report significantly higher rates than in the general population, with 40.2% [31], 51.9% [32], 68% [39] and 79% [23], respectively (Fig. 2).

### Availability of DPW

**Fig. 3:**
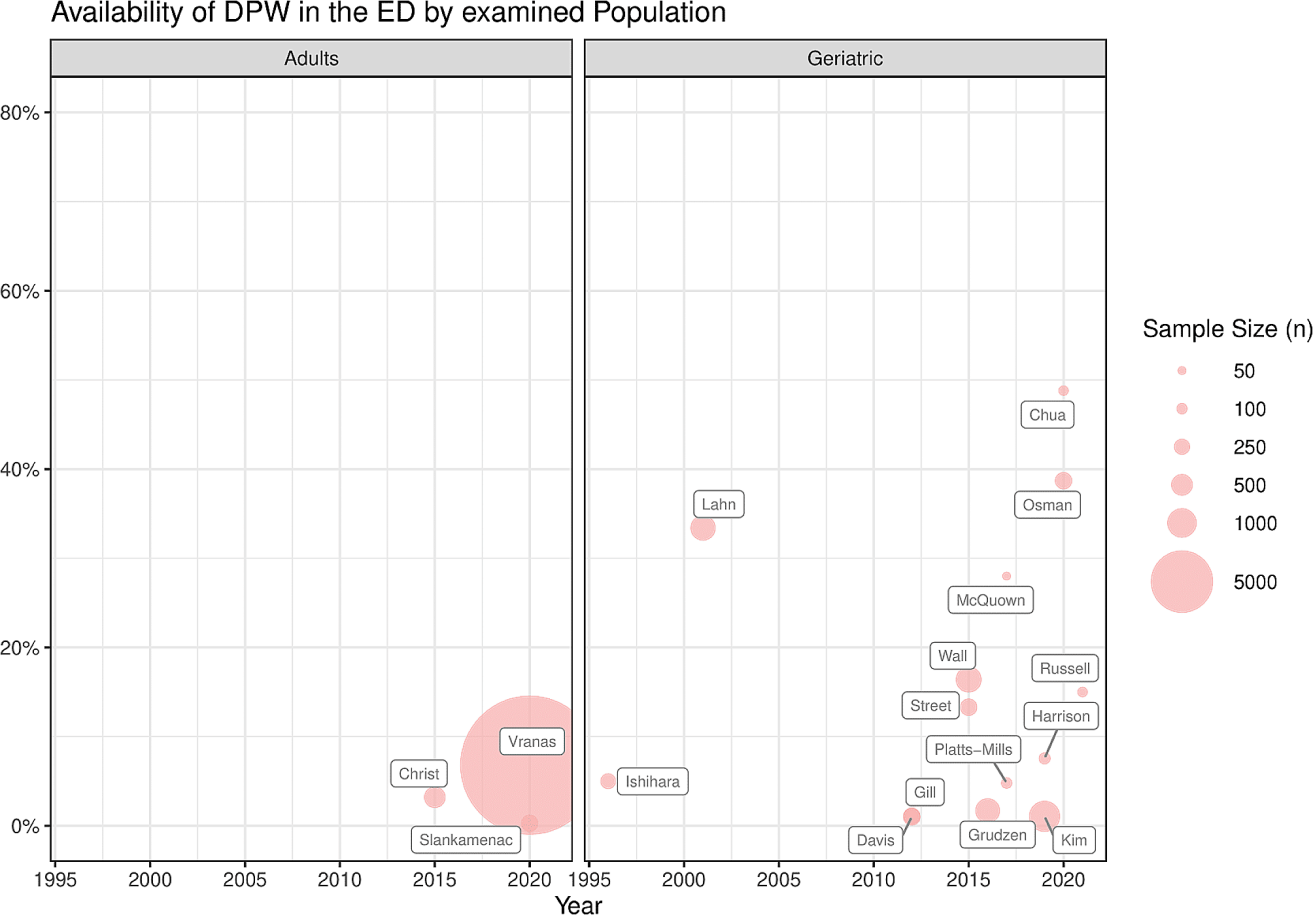
DPW availability by study population (left: adult, right: geriatric) and year of publication

In the adult population of ED patients, the percentage of patients with DPW available in the ED was between 0.3% [36] and 6.8% [37] (Fig. 3). Availability in the geriatric population varied broadly between 1.1% [26, 34] and 48.8% [35] (Fig. 3).

In the studies where both existence and availability were examined, the proportion of existing DPW that were available was calculated. Five studies reported values of 11.6% or below [26–28, 31, 32, 36]. Russell et al. found that in patients with terminal disease, 41.7% of possessed DPW were available upon ED presentation [38]; McQuown and Harrison reported 41.2% and 50% of DPW being available in NH patients transferred to the ED, respectively [33, 39]. One outlier, Street et al., found that for NH residents who possessed a DPW, it was available for 97.6% in the ED [29].

### Predictors of DPW existence and/or availability

All studies that examined potential predicting factors [5, 21, 22, 24, 28, 30–32, 36], except one [39] identified older age as a positive predictor of documented patient wishes Likewise, several studies found a correlation between comorbidities or self-rating of generally poorer health and increased likelihood of holding a DPW [22, 29, 36]. The use of health care services, especially NH or similar institutions, seems to correlate with a higher rate of DPW [29, 30, 36]. In a 1:1 matched cohort study (150 NH residents with 150 independently living persons of the same age) all DPWs were in the NH group [29].

Having a primary care physician was found once as a positive predictor [22], and once as a negative [28]. Various other socio-demographic variables with positive influence were identified, for example: female gender [5, 20, 30], having children [22], being in a relationship [28] and higher level of education [24], as well as ethnicity defined as “white” [5, 22, 30, 32] (Supplementary Tab. s5). Most of the studies did not calculate effect sizes, thus, further evaluation and analysis was not feasible.

## Discussion

Here, we present a systematic and comprehensive overview of the literature until September 2023 addressing DPW in ED settings in the geriatric and non-geriatric population. We searched five databases and two reviewers screened abstracts and full-text manuscripts following predefined inclusion and exclusion criteria. A risk of bias assessment was performed for all included studies. A descriptive analysis of all studies was conducted.

In the general population of patients presenting to the ED, one in five to one in four patients reported having a DPW, but the actual availability in the ED was much lower by comparison with no more than 7%. In geriatric populations, both existence and availability rates were highly variable: Existence rates were as high as 70%, whereas availability rates were below 50% in all studies. In addition to already known predictors, such as age and health status, further variables influencing rates of DPW were identified, such as social structure, institutional factors in nursing homes, patient education in primary care and previous hospital admissions in the last 12 months including intensive care treatment.

### Existence, availability and predictors

The first available data on DPW in the general ED population were published more than 25 years ago, with reported rates of 22–27% [20, 21] Despite increased efforts for education in recent years [32], more recent studies from 2012 to 2021 did not show a clear upward trend in rates of DPW existence, which ranged from 20 to 28% [27, 28, 36]. A possible increasing effect of the COVID-19 pandemic was shown in other contexts [40], however all studies included in this review only presented data from pre-pandemic times.

The high variability of existence and availability rates in the geriatric population could be explained by heterogeneity of in- and exclusion criteria in different studies (e.g., geriatric defined in several studies by age ≥ 60, ≥65, ≥ 70 or ≥ 75 years [11, 20, 24, 26, 29, 31–33], in others by being NH residents [4, 23, 25, 30, 34, 35, 39]) as well as institutional circumstances (e.g., in one Australian study the nursing home was particularly well integrated with the hospital [35]). One study showed that this variance is not only inter-individual but also inter-institutional: availability rates were 0% in some nursing homes, 94% in others [5].

Of note, availability rates of DPW in the emergency department were distinctly lower than possession rates in all respective studies; affecting both geriatric and comparatively younger populations alike. Some studies found no more than 5% of existing DPW were actually available on presentation to emergency clinicians [26, 27, 31, 36].

Studies which reported comparably high existence and availability rate, originated mainly from the United States and Australia [23, 32, 35, 39]. This may partly be attributed to several initiatives aimed at increasing the number of DPW in nursing homes in Australia since 2007 [29] or such as the *Choosing Wisely campaign* in the US [15].

Among the predictors identified, some were related to socioeconomic factors, such as education. Similar findings have previously been reported regarding the general availability and completion of advance care directives in the community [41]. It can be hypothesized that the socioeconomic background influences the likelihood of DPW existence, which might be due to an influence of levels of awareness of palliative care and advance directives by race, ethnicity and socioeconomic variables e.g. in different Californian populations [42].

Remarkably, fewer studies than expected identified having a general practitioner (GP) as a positive predictor. One study even found it to be a negative predictor [20]. Conversely, having a specialist physician was consistently found to be a positive predictor, which might be confounded by higher morbidity (already identified as a positive predictor) when being in the need of a specialist physician.

We hypothesize that the general possibility of documenting patient wishes and the available options to do so is not known well enough in both patients and medical staff. Moreover, especially younger and healthier people may be reluctant to address preemptive decisions concerning significant sickness or their own death. Furthermore, patients already possessing DPW often don’t bring them to the ED. This may be corroborated by Emergency Medical Services (EMS) not asking or checking for DPW very often before transporting patients to the ED, as indicated by Harrison and Vranas [33, 37].

According to this data, evaluating goals of care through DPW currently is rarely feasible in an ED setting, although essential. In the ED, a potential approach to increase both availability and patients’ awareness of DPWs would be the implementation of mandatory inquiry and documentation as part of standard operating procedure. In an outpatient setting, increased numbers of qualified low-threshold ACP advice services might reduce barriers for patients willing to document their preferences. Moreover, a visit to the ED, treatment in hospital or ICU seems to increase the willingness and demand to document one’s wishes, additionally confirmed by the increase of DPW possession rate after a 60-day-period simply due to the contact with this topic shown in one study [36], which offers a possibility to ED medical staff to at least give an impulse and start the conversation. Finally, more research also including qualitative studies is needed to establish further causes for patients choosing not to have a DPW.

### Limitations

One limitation is the strong heterogeneity of the included studies regarding time period, country, sample size, population, documentation, endpoints and concepts of DPW, which limits comparability. This is further complicated by regional differences in the definition of DPW (e.g., one study classified AD separately from DNR [5]), even though these were accounted for in this analysis.

The use of “general population” and “geriatric population” as categories is not entirely consistent between studies. Therefore, some overlap between the two groups is present.

We acknowledge that visual depiction of descriptive analysis to facilitate comparison between studies has limitations, however due to the heterogeneity of the studies, the data set was not amenable to meta-analysis or meta-regression.

To the best of our knowledge the only other systematic review on this topic [43] analyzed six studies from the US [4, 20–22, 27, 31], all of which were also included in our review. The authors describe comparable results of patient-reported AD possession rates for the general population ranging between 20% and 27%. However, the results of one study [20] were likely misreported by this review as “53%” instead of “22% (n = 53)” in the original publication, probably due to an error in data entry. Results and conclusions for the older cohorts might be affected by this.

## Conclusions

DPW possession rates and availability were low among ED patients. Thus, public awareness campaigns, intensified education of medical staff and improved cooperation between GPs, NHs and hospitals is needed to address this unmet medical need. ED staff could also play a vital role by initiating conversations about patient wishes. (Inter-)Nationally standardized forms of documentation and new modalities for saving and accessing such information (e.g., emergency cards or digital health passes) might aid in increasing availability. More research is required to structurally explore the efficacy of such measures, as well as to assess the contents and effects of existing DPW and their concordance with therapeutic decisions being made.

### Electronic supplementary material

Below is the link to the electronic supplementary material.

Supplementary Material 1

## Data Availability

The datasets generated and analyzed for this systematic review were extracted from the original publications cited. The underlying datasets of each cited publication were– unless published– not otherwise available. All cited publications are available from the corresponding author on reasonable request.

## Abbreviations

ACP: Advance care planning
AD: Advance directive
DNI: Do not inubate
DNR: Do not resuscitate
DPW: Documentation of patient whishes
ED: Emergency department
LW: Living will
NH: Nursing home

## References

[1] Spickermann M, Lenz P (2018). Early) Palliative Care in Rettungsdienst Und Notaufnahme. DMW - Dtsch Med Wochenschr.

[2] Smith AK, McCarthy E, Weber E, Cenzer IS, Boscardin J, Fisher J (2012). Half of older americans seen in emergency department in last month of life; most admitted to hospital, and many die there. Health Aff Proj Hope.

[3] Siegel M, Bigelow S (2018). Palliative Care Symptom Management in the Emergency Department: the ABC’s of Symptom Management for the emergency physician. J Emerg Med.

[4] Lahn M, Friedman B, Bijur P, Haughey M, Gallagher EJ (2001). Advance directives in skilled nursing facility residents transferred to emergency departments. Acad Emerg Med off J Soc Acad Emerg Med.

[5] George N, Barrett N, McPeake L, Goett R, Anderson K, Baird J (2015). Content validation of a Novel Screening Tool to identify Emergency Department patients with significant Palliative Care needs. Acad Emerg Med.

[6] Schelling P (2016). [Under scrutiny by the state prosecutor: legal pitfalls in emergency medicine]. Anaesthesist.

[7] McEwan A, Silverberg JZ (2016). Palliative Care in the Emergency Department. Emerg Med Clin North Am.

[8] Lowery DS, Quest TE (2015). Emergency medicine and palliative care. Clin Geriatr Med.

[9] George N, Phillips E, Zaurova M, Song C, Lamba S, Grudzen C (2016). Palliative Care Screening and Assessment in the Emergency Department: a systematic review. J Pain Symptom Manage.

[10] Hartog CS, Spies CD, Michl S, Janssens U (2020). Advance Care Planning in Zeiten Der Corona-Pandemie– Eine Chance für die Patientenautonomie in Der Akutsituation. Med Klin - Intensivmed Notfallmedizin.

[11] Osman AD, Rahman MA, Lam L, Lin CC, Yeoh M, Judkins S (2020). Cardiopulmonary resuscitation and endotracheal intubation decisions for adults with advance care directive and resuscitation plans in the emergency department. Australas Emerg Care.

[12] Nauck F, Marckmann G, Schmitten J (2018). Der. Behandlung Im Voraus planen– bedeutung für die Intensiv- Und Notfallmedizin. AINS - Anästhesiol · intensivmed ·. Notfallmedizin · Schmerzther.

[13] Kupensky D, Hileman BM, Emerick ES, Chance EA (2015). Palliative Medicine Consultation reduces length of Stay, improves Symptom Management, and clarifies advance directives in the geriatric Trauma Population. J Trauma Nurs.

[14] Mogul AS, Cline DM, Gabbard J, Bryant C (2019). Missed opportunities: integrating Palliative Care into the Emergency Department for older adults presenting as Level I triage Priority from Long-Term Care facilities. J Emerg Med.

[15] Grudzen CR, Richardson LD, Johnson PN, Hu M, Wang B, Ortiz JM (2016). Emergency Department-Initiated Palliative Care in Advanced Cancer: a Randomized Clinical Trial. JAMA Oncol.

[16] Moher D, Liberati A, Tetzlaff J, Altman DG, PRISMA Group (2009). Preferred reporting items for systematic reviews and meta-analyses: the PRISMA statement. PLoS Med.

[17] Shamseer L, Moher D, Clarke M, Ghersi D, Liberati A, Petticrew M (2015). Preferred reporting items for systematic review and meta-analysis protocols (PRISMA-P) 2015: elaboration and explanation. BMJ.

[18] Bramer WM, Rethlefsen ML, Kleijnen J, Franco OH (2017). Optimal database combinations for literature searches in systematic reviews: a prospective exploratory study. Syst Rev.

[19] Munn Z, Moola S, Lisy K, Riitano D, Tufanaru C (2015). Methodological guidance for systematic reviews of observational epidemiological studies reporting prevalence and cumulative incidence data. Int J Evid Based Healthc.

[20] Ishihara KK, Wrenn K, Wright SW, Socha CM, Cross M (1996). Advance directives in the emergency department: too few, too late. Acad Emerg Med off J Soc Acad Emerg Med.

[21] Llovera I, Mandel FS, Ryan JG, Ward MF, Sama A (1997). Are emergency department patients thinking about advance directives?. Acad Emerg Med off J Soc Acad Emerg Med.

[22] Llovera I, Ward MF, Ryan JG, Lesser M, Sama AE, Crough D (1999). Why don’t emergency department patients have advance directives?. Acad Emerg Med off J Soc Acad Emerg Med.

[23] Saliba D, Kington R, Buchanan J, Bell R, Wang M, Lee M (2000). Appropriateness of the decision to transfer nursing facility residents to the hospital. J Am Geriatr Soc.

[24] Taylor DM, Ugoni AM, Cameron PA, McNeil JJ (2003). Advance directives and emergency department patients: ownership rates and perceptions of use. Intern Med J.

[25] Carter L, Skinner J, Robinson S (2009). Patients from care homes who attend the emergency department: could they be managed differently. Emerg Med J EMJ.

[26] Gill GGK, Fukushima E, Abu-Laban RB, Sweet DD (2012). Prevalence of advance directives among elderly patients attending an urban Canadian emergency department. CJEM.

[27] Davis CP (2012). Emergency department visits: we are not prepared. Am J Emerg Med.

[28] Christ M, Liebeton J, Breker IM, Grett M, von Auenmüller KI, Trappe HJ (2015). [The availability of living wills in an interdisciplinary emergency department: results of a patient survey]. Dtsch Med Wochenschr 1946.

[29] Street M, Ottmann G, Johnstone MJ, Considine J, Livingston PM (2015). Advance care planning for older people in Australia presenting to the emergency department from the community or residential aged care facilities. Health Soc Care Community.

[30] Wall J, Hiestand B, Caterino J (2015). Epidemiology of Advance directives in Extended Care Facility patients presenting to the Emergency Department. West J Emerg Med.

[31] Grudzen CR, Buonocore P, Steinberg J, Ortiz JM, Richardson LD, Aslakson RA (2016). Concordance of Advance Care Plans with Inpatient directives in the Electronic Medical Record for older patients admitted from the Emergency Department. J Pain Symptom Manage.

[32] Platts-Mills TF, Richmond NL, LeFebvre EM, Mangipudi SA, Hollowell AG, Travers D (2017). Availability of Advance Care Planning Documentation for older Emergency Department patients: a cross-sectional study. J Palliat Med.

[33] Harrison L, O’Connor E, Renner CH, Kluesner N (2019). Assessing the accessibility to the IPOST at admission to the Emergency Department. Am J Emerg Med.

[34] Kim K, Lee DH, Yune HY, Wee JH, Kim DH, Kim EC (2019). Identifying potentially avoidable Emergency Department visits of long-term Care Hospital residents in Korea: a Multicenter Retrospective Cohort Study. BioMed Res Int.

[35] Chua TH, Foong JRJ, Tan RR, Rukasha PN, Hullick C (2020). Assessment of advance care planning documentation for residents of residential aged care facilities presenting to the emergency department. Aust Health Rev.

[36] Slankamenac K, Rütsche N, Keller DI (2020). Availability of advance directives in the emergency department. Swiss Med Wkly.

[37] Vranas KC, Lin AL, Zive D, Tolle SW, Halpern SD, Slatore CG (2020). The Association of POLST with intensity of treatment among patients presenting to the Emergency Department. Ann Emerg Med.

[38] Russell E, Hall AK, McKaigney C, Goldie C, Harle I, Sivilotti MLA (2021). Code Status Documentation availability and accuracy among emergency patients with end-stage disease. West J Emerg Med.

[39] McQuown CM, Frey JA, Amireh A, Chaudhary A (2017). Transfer of DNR orders to the ED from extended care facilities. Am J Emerg Med.

[40] Auriemma CL, Halpern SD, Asch JM, Van Der Tuyn M, Asch DA (2020). Completion of advance directives and documented Care preferences during the Coronavirus Disease 2019 (COVID-19) pandemic. JAMA Netw Open.

[41] Rao JK, Anderson LA, Lin FC, Laux JP (2014). Completion of advance directives among U.S. consumers. Am J Prev Med.

[42] Bazargan M, Cobb S, Assari S, Kibe LW (2021). Awareness of Palliative Care, Hospice Care, and Advance directives in a racially and ethnically diverse sample of California adults. Am J Hosp Palliat Care.

[43] Oulton J, Rhodes SM, Howe C, Fain MJ, Mohler MJ (2015). Advance directives for older adults in the emergency department: a systematic review. J Palliat Med.

