# Supplementary material for: Advance directives in the emergency department–a systematic review of the status quo
Source: BMC Health Serv Res. 2024 Apr 3;24:426. doi: 10.1186/s12913-024-10819-1 (PMC10993583; doi:10.1186/s12913-024-10819-1)
Supplement: Supplementary file 1 — Supplementary Material 1 [file 12913_2024_10819_MOESM1_ESM.docx]

**Advance Directives in the Emergency Department – a Systematic Review of the Status Quo** *Supplementary*

***Tab.s1:*** *Searched Databases and Search Algorithms used. Title/Abstract/Keywords for Cochrane, Web of Science and Embase; Title for Google Scholar; MeSH-Terms for MEDLINE.*

| **Database** | **Search Algorithm** |
| --- | --- |
| **Cochrane** | (("advance directives"):ti,ab,kw OR ("dnr order"):ti,ab,kw OR ("do not resuscitate"):ti,ab,kw OR ("living will"):ti,ab,kw ) AND ( (emergency department):ti,ab,kw OR (emergency room):ti,ab,kw OR (emergency services):ti,ab,kw) |
| **Web of Science** | (TS=("advance directives") OR TS=("living will") OR TS=("DNR order") OR TS=("do not resuscitate")) AND (TS=(emergency department) OR TS=(emergency room) OR TS=(emergency services)) |
| **Embase via Ovid** | ("advance directives" OR "living will" OR "dnr order" OR "do not resuscitate") AND ("emergency department" OR "emergency room" OR "emergency services") |
| **Google Scholar** | "advance directives" OR "living will" OR "dnr order" OR "do not resuscitate" "emergency department" OR "emergency room" OR "emergency services" |
| **MEDLINE (Pubmed)** | ((("Advance Directives"[Mesh Terms]) OR ("Advance Care Planning"[Mesh Terms]) OR ("Living Wills"[Mesh Terms]) OR ("Resuscitation Orders"[Mesh Terms])) AND (emergency services, hospital [MeSH Terms])) OR ((((("Advance Directives/statistics and numerical data"[MAJR]) OR (("Advance Directives"[Mesh Terms]) AND ("Availability" OR "available")))) OR (("Advance Care Planning/statistics and numerical data"[MAJR]) OR (("Advance Care Planning"[Mesh Terms]) AND ("Availability" OR "available"))))) AND (emergency services, hospital [MeSH Terms]) NOT (casereports [Filter]))) |

| ***Tab. s2:*** *Publications excluded upon full-text assessment.* | | |
| --- | --- | --- |
| **Author** | **Year** | **Exclusion Criterion** |
| Balentine | 1996 | DPW issued after ED contact |
| Chang | 2021b |  |
| Chen | 2019 |  |
| Cheng | 2016 |  |
| Cheng | 2019 |  |
| Miller | 2021 |  |
| Richardson | 2013 |  |
| Su | 2018 |  |
| Wren | 1992 |  |
| Wong | 2016 |  |
| Alsaati | 2020 | No Primary Prevalence Data on DPW |
| Balakrishnan | 2019 |  |
| Brunner | 2014 |  |
| Carter | 2020 |  |
| Chang | 2021a |  |
| Cheng | 2015 |  |
| de Groot | 2012 |  |
| Dexter | 2003 |  |
| Elsayem | 2017 |  |
| Ermers | 2021 |  |
| Ferdinando | 2019 |  |
| Grant | 1994 |  |
| Grautoff | 2022 |  |
| Hughes | 2021 |  |
| Manifold | 1999 |  |
| Miller | 1996 |  |
| Mogul | 2019 |  |
| Ouchi | 2017 |  |
| Ouchi | 2019 |  |
| Oulton | 2015 |  |
| Padberg | 2014 |  |
| Pajka | 2021 |  |
| Pimsen | 2022 |  |
| Rajda | 2015 |  |
| Reid | 2013 |  |
| Richardson | 2014 |  |
| Riley | 2004 |  |
| Rincon | 2011 |  |
| Roden-Foreman | 2017 |  |
| Rolland | 2021 |  |
| Schreiber | 1998 |  |
| Siegrist | 2018 |  |
| Trzeciak | 2006 |  |
| Vancini-Campanharo | 2017 |  |
| Walsh-Kelly | 1999 |  |
| Wang | 2021 |  |
| Weinick | 2008 |  |
| Weng | 2017 |  |
| Wrenn | 1992 |  |
| Jessen | 2021 |  |
| Webster | 2021 |  |
| Sivayoham | 2023 |  |
| Weinick | 2008 |  |
| Adams | 2013 | Population inadequate (referred to only a subset of ED patients defined by specialty (e.g., only dept. of surgery), illness (e.g., only sepsis) or patient disposition (e.g., only out- or inpatient)) |
| Antolín | 2010 |  |
| Asiaban | 2023 |  |
| Ballou | 2019 |  |
| Boonmee | 2020 |  |
| Brokmann | 2014 |  |
| Chongthanadon | 2023 |  |
| Dean | 2015 |  |
| Ding | 2020 |  |
| Drumheller | 2016 |  |
| Fan | 2017 |  |
| Gaw | 2012 |  |
| Graw | 2021 |  |
| Haddad | 2021 |  |
| Han | 2007 |  |
| Hanson | 1994 |  |
| Harb | 2021 |  |
| Hemphill | 2004 |  |
| Hirvonen | 2018 |  |
| Hsu | 2014 |  |
| Hsu | 2022 |  |
| Khan | 2007 |  |
| Kim | 2022 |  |
| Kupensky | 2015 |  |
| Lee | 2022 |  |
| Marco | 2015 |  |
| Matsushima | 2016 |  |
| Mills | 2016 |  |
| Moman | 2017 |  |
| Monsomboon | 2022 |  |
| Nathens | 2008 |  |
| Olds | 2023 |  |
| Powell | 2013 |  |
| Puchongmart | 2023 |  |
| Pyles | 2022 |  |
| Rahman | 2001 |  |
| Salottolo | 2015 |  |
| Sarkari | 2016 |  |
| Wade | 2013 |  |
| Wang | 2020 |  |
| Wu | 2021 |  |
| Battisti | 2020 |  |
| Richardson | 2015 |  |
| Richardson | 2013 |  |

| *Tab. s3: Overview of included studies, sorted by year of publication.* | | | | | | | | | | | |
| --- | --- | --- | --- | --- | --- | --- | --- | --- | --- | --- | --- |
|  | **Time, Setting** | **Inclusion / Exclusion Criteria** | **A/G** | **n** | **Age [years]** | **Fe-male** | **Coun-try** | **Methods** | **DPW Definition** | **DPW Ex-istence** | **DPW Avai-** **lability** |
| Ishihara 1996 | 1994, academic ED, 24/7 | **I:** ≥65 Y. or severe chronic illness  **E:** SB, ICQ | G | 238 | M 67  (IQR 51-75) | 57% | US | Prospective, convenience sample, oral survey | AD reported | 22.3% | 5.0% (22.6%) |
| Llovera 1997 | 1995/96, academic ED, 24/7 | **I:** ≥18 Y.  **E:** ICQ, SB | A | 511 | ∅ 53  (SD ±20) | 52.1% | US | Prospective, convenience sample, written survey | AD reported | 27% | -/- |
| Llovera 1999 | 1997, ED, represen-tative shifts | **I:** ≥18 Y.  **E:** ICQ, SB, RC | A | 476 | ∅ 49  (SD ±20) | 50% | US | Prospective, written Survey | AD reported | 23.5% | -/- |
| Saliba 2000 | 1994-95,  13 NHs | **I:** NH residents transferred to ED per NH lists, ran-domly 16 per NH **E:** scheduled >24h transfer, records not found, evaluation of newly documen-ted hip fracture | G | 100 | ∅ 81.6  (SD ±10.6) | 68% | US | Retrospective record review | AD information in "minimum data set” transferred from NH to ED | 79% | -/- |
| Lahn 2001 | No year, 2 urban EDs | **I:** NH residents  **E:** none | G | 715 | ∅ 79  (SD ±13) | 65% | US | Prospective, record review | AD/DNR available (HCP excluded by us) | -/- | 33.4% |
| Taylor 2003 | 2001, academic ED, weekdays, by daytime | **I:** ≥60 Y.  **E:** ICQ, SB | G | 403 | M 73.3  (IQR 60-91) | 40.7% | AU | Prospective, convenience sample, written survey | AD reported (HCP excluded by us) | 7.9% | -/- |
| Carter 2009 | 2007 | **I:** NH residents | G | 114 | ∅ 83.8  (SD ±10.3) | 71.5% | UK | Prospective, record review | "resuscitation status noted" | 2.6% | -/- |
| Gill 2012 | 2008, academic ED, by daytime | **I:** ≥70 Y., CC+  **E:** ICQ without proxy available | G | 280 | ∅ 80.6 | 55% | CA | Prospective, written survey | AD reported / available | 19.3% | 1.1% (5.6%) |
| Davis 2012 | 2011, ED, by daytime | **I:** Treated by study physician  **E:** none | A | 309 | M 60 | 47% | US | Prospective,  oral survey | AD reported / available | 21% | 1% (4.6%) |
| Street 2015 | 2011, 3 EDs | **I:** ≥65 Y.  **E:** none | G | 300 | ∅ 86.2  (SD ±6.5) | 67.7% | AU | Retrospective matched cohort study, record review | ACP reported / available | 13.7% | 13.3% (97.6%) |
| Christ 2015 | 2014, ED | **I:** Hospitalized, ≥18 Y.  **E:** Pregnant, legal proxy, RC, critically ill | A | 496 | ∅ 64.9  (SD ±18.8) | 53.8% | DE | Prospective, oral survey | AD reported / available | 27.8% | 3.2% (11,6%) |
| Wall 2015 | 2007-2009 | **I:** Arriving from NH **E:** return visits, <18 Y. | G | 754 | M 66  (IQR 54-78) | 53.4% | US | Retrospective record review | DNR-order (regardless of content) |  | 16.4% |
| Grudzen 2016 | 2012, adult & geriatric ED | **I:** ≥65 Y., ESI >1, able to walk, CC+  **E:** SB, CI | G | 682 | ∅ 75.8  (SD ±8.0) | 58.1% | US | Prospective, convenience sample, oral survey + record review | AD reported or in records | 40.2% | 1.7% (4.4%) |
| McQuown 2017 | 2012-2013, urban level 1 trauma center ED, 10:00-15:00 | **I:** Arriving from NH via ambulance **E:** Seen by study teem while working clinically, major trauma, transfer from other hospital | G | 50 | ∅ 78 | 56% | US | Prospective, record review | Ohio DNR order | 68% | 28% (41.2%) |
| Platts-Mills 2017^1^ | 2016, academic ED | **I:** ≥75 Y. or ≥65. Y. with high risk of death  **E:** SB, CI | G | 104 | Not reported | 44.2% | US | Prospective, oral Survey + record review | AD reported (DNR, POLST and HCP excluded by us) | 51.9% | 4.8% (9.3%) |
| Harrison 2019 | 2017, level 1 trauma center ED | **I:** ≥65 Y. arriving by ambulance, interfacility transport or pirvate vehicle | G | 119 | ∅ 80 | -/- | US | Prospective, record review | IPOST | 15.13% | 7.56% (50%) |
| Kim 2019 | 2017, 5 academic EDs | **I:** ≥65 Y from NH | G | 1.131 | Reported In categories | 54.11% | KR | Retrospectiverecord review | “DNR order signed at NH before transfer” | -/- | 1.06% |
| Slankamenac 2020 | 2017, academic ED, 24/7 | **I:** ≥18 Y. **E:** SB, CI, no time availbale, analphabetism, critically ill | A | 292 | M 46  (IQR 29-63) | 49.3% | CH | Prospective, written survey + 60-day follow-up via e-mail | AD reported / available | 19.9% | 0.3%  (1,7%) |
| Chua 2020 | 2018, ED | **I:** NH residents >75 Y.  **E:** none | G | 80 | ∅ 87.9  (SD ±4.7) | 61.7% | AU | Prospective, record review | ACP available | -/- | 48.8% |
| Osman 2020 | 2019, ED | **I:** ≥65 Y.  **E:** none | G | 300 | ∅ 78  (SD ±8.1) | 50.7% | AU | Retrospectiverecord review | ACP presence (ambiguous) | -/- | 38.7% |
| Vranas 2020 | 2015-2016, academic ED | **I:** triaged urgent, emergent or life-threatening **E:** >10 ED visits in study period, left AMA, transfer to other facility, left without seen, unknown | A | 26.128 | ∅ 66.1  (SD 15.5) | 50.33% | US | Retrospective cohort study | POLST registry | -/- | 6.8% |
| Russell 2021 | 2016, academic ED | **I:** Palliative care consultation in prior 3 months or metastatic malignancy or home O2 use for COPD/heart failure or dialysis for CKD or progressive neurodegenera-tive disease (incl. dementia) | G | 82   (of those 32 interviewed) | M 78  (IQR 67–86) | 55% | CA | Convenience sample | Possession = EHR; Availability = accompanying paper docu-mentation | 36% | 15% (41.7%) |

***Columns:*** *1. Study; 2. Time period and setting; 3. Inclusion (I) and Exclusion (E) criteria; 4. Classification as adult population (A) or geriatric population (G); 5. Sample size (n); 6. ∅ age with standard deviation (SD) or Median with inter quartile range (IQR); 7. Percentage of female subjects; 8. Country; 9. Study design and methodology; 10. Definition of DPW used; 11. Rate of having DPW; 12. Rate of DPW being available at ED as percentage of the overall sample and (in brackets) of patients possessing DPW*

***Abbreviations:*** *Median (M); Nursing Home (NH); Emergency Department (ED); Speech barrier (SB); Cognitive impairment (CI); Inability to complete questionnaire (ICQ); Reduced level of consciousness (RC); Capability to consent (CC+); Emergency Severity Index (ESI); Manchester Triage Score (MTS), Physician Orders for Life-Sustaining Treatment, a form of advance care planning used in the US (POLST).*

*^1^: Platts-Mills et al. reported possession rates for AD, DNR, POLST and HCP separately, without being mutually exclusive. Therefore, no exact possession rate can be calculated for the category [AD, DNR or POLST]; the true value must lie between 51.9% and 58.7%. The lowest extreme is assumed in the following.*

| ***Tab. s4:*** *Risk of Bias Assessment with Items in accordance with Joanna Briggs Institute (JBI) Clinical Appraisal Checklist for Prevalence Studies.* | | | | | | | | | |
| --- | --- | --- | --- | --- | --- | --- | --- | --- | --- |
|  | **1** | **2** | **3** | **4** | **5** | **6** | **7** | **8** | **9** |
| **Ishihara**  **1996** | Yes | Yes | No | Yes | NA | NA | NA | Yes | NA |
| **Llovera**  **1997** | Yes | No | Yes | Yes | NA | NA | NA | Yes | Yes |
| **Llovera**  **1999** | Yes | No | Yes | Yes | NA | NA | NA | Yes | Yes |
| **Saliba**  **2000** | Yes | Unclear | No | Yes | NA | NA | NA | Yes | NA |
| **Lahn**  **2001** | Yes | Yes | Yes | Yes | NA | NA | NA | Yes | NA |
| **Taylor**  **2003** | Yes | Yes | Yes | Yes | NA | NA | NA | Yes | NA |
| **Carter**  **2009** | Yes | Unclear | No | Yes | NA | NA | NA | Yes | NA |
| **Davis**  **2012** | Yes | Yes | Yes | Yes | NA | NA | NA | Yes | NA |
| **Gill**  **2012** | Yes | Yes | No | Yes | NA | NA | NA | Yes | NA |
| **Christ**  **2015** | Yes | Yes | Yes | Yes | NA | NA | NA | Yes | NA |
| **Street**  **2015** | Yes | Yes | Yes | Yes | NA | NA | NA | Yes | NA |
| **Wall**  **2015** | Yes | Yes | Yes | Yes | NA | NA | NA | Yes | NA |
| **Grudzen**  **2016** | No | Yes | Yes | Yes | NA | NA | NA | Yes | NA |
| **McQuown**  **2017** | Yes | Unclear | No | Yes | NA | NA | NA | Yes | NA |
| **Platts-Mills**  **2017** | No | Yes | No | Yes | NA | NA | NA | Yes | NA |
| **Harrison**  **2019** | Yes | Unclear | No | No | NA | NA | NA | No | Unclear |
| **Kim**  **2019** | Yes | Yes | Yes | Yes | NA | NA | NA | Yes | NA |
| **Chua**  **2020** | Yes | Yes | No | Yes | NA | NA | NA | Yes | NA |
| **Osman**  **2020** | Yes | Yes | Yes | Yes | NA | NA | NA | Yes | NA |
| **Slankamenac**  **2020** | Yes | Yes | Yes | Yes | NA | NA | NA | Yes | NA |
| **Vranas**  **2020** | Yes | No | Yes | Yes | NA | NA | NA | Yes | NA |
| **Russell**  **2021** | Yes | Yes | No | Yes | NA | NA | NA | Yes | NA |
| ***Checklist Items:***   1. *Was the sample frame appropriate to address the target population?* 2. *Were study participants sampled in an appropriate way?* 3. *Was the sample size adequate?* 4. *Were the study subjects and the setting described in detail?* 5. *Was the data analysis conducted with sufficient coverage of the identified sample?* 6. *Were valid methods used for the identification of the condition?* 7. *Was the condition measured in a standard, reliable way for all participants?* 8. *Was there appropriate statistical analysis?* 9. *Was the response rate adequate, and if not, was the low response rate managed appropriately?* | | | | | | | | | |

| *Tab. s5: Predictors with positive, none or negative correlation with existence or availability of DPW.* | | | | |
| --- | --- | --- | --- | --- |
| Predictor | | positive | none | negative |
| SOCIODEMOGRAPHIC ASPECTS | | | | |
| Age | Higher Age | Wall, George, Llovera 1999, Llovera 1997, Platts, Taylor, Christ, Slank | McQuown |  |
| Gender | Female | George, Ishihara, Wall | Llovera 1997, Llovera 1999, McQuown |  |
| Ethnicity | “White” (vs. “Non-White”) | George, Llov99, Platts-Mills, Wall |  |  |
|  | “African American” (vs. “Hispanic”) | Llovera 1999 |  |  |
| Religion | Catholic |  |  | Llovera 1999 |
| Education | Higher Education | Taylor | Slankamenac |  |
| Civil status | Single |  |  | Slankamenac |
|  | Widdowed | Llovera 1999, Slankamenac |  |  |
|  | Having children | Llovera 1999 | Slankamenac |  |
| Geography | Nationality of country, the study was in (CH) | Slankamenac |  |  |
|  | Born in the country, the study was in (AU) | Taylor |  |  |
| MEDICAL ASPECTS | | | | |
| Comorbi-dities | Comorbidities in general | Llovera 1999, Slankamenac | Ishihara |  |
|  | Neurodegenerative disease | Street |  |  |
|  | Malignant disease | Slankamenac |  |  |
|  | Charlson Comorbidity Score | Street |  |  |
|  | Life-threatening comorbidity (self-assessed) | Llovera 1999 |  |  |
|  | General health “bad” (self-assessed) | Llovera 1997, Taylor |  |  |
| ED Presentation | Arriving via ambulance | Christ |  |  |
|  | Accompanied by someone to the ED |  |  | Street |
| HEALTH CARE SYSTEMIC ASPECTS | | | | |
| Hospital | Number of hospital days in the last 12 months | Slankamenac |  |  |
|  | Admitted from ED to inpatient unit | Wall |  |  |
| Other ACP Documents | Having Health Care Power of Attorney | Wall |  |  |
|  | Having Living WIll | Wall |  |  |
| Nursing Care | Nursing home residents | Street |  |  |
|  | Supervised living situation | Slankamenac |  |  |
| Outpatient Care | Having a primary care physician | Llovera 1997,  Llovera 1999 | Platts-Mills | Slankamenac |
|  | Having a primary care physician, connected to the study institution | Platts-Mills |  |  |
|  | Having a specialist physician | Llovera 1999, Slankamenac |  |  |
